# Supplementary material for: Integrated analysis of the aging brain transcriptome and proteome in tauopathy
Source: Mol Neurodegener. 2020 Sep 29;15:56. doi: 10.1186/s13024-020-00405-4 (PMC7526226; doi:10.1186/s13024-020-00405-4)
Supplement: Supplementary file 1 — Additional file 1: Supplemental Figures. Figure S1. Expression of TauWT and TauR406W in transgenic flies. Figure S2. Venn diagrams showing overlaps in differential expressed genes. Figure S3. Potential impact of missingness on proteome analyses. Figure S4. Reverse transcription-quantitative PCR validation of differentially expressed genes. Figure S5. Western blot validation of differentially expressed genes from LC-MS/MS proteomics. Figure S6. Age and Tau are major drivers of gene expression differences among samples. Figure S7. TauWT-triggered differential expression in the transcriptome vs. proteome. Figure S8. Additional examples of differentially expressed transcripts and proteins in TauR406W flies. Figure S9. Overlap between TauR406W- and aging-induced gene expression changes. Figure S10. Weighted gene coexpression network analysis in TauR406W flies. Figure S11. Integration of Tau-induced transcriptome and proteome changes in gene expression clusters. Figure S12. Non-negative matrix factorization rank survey for unsupervised hierarchical clustering. Figure S13. WGCNA cluster dendrograms. [file 13024_2020_405_MOESM1_ESM.pdf]

ADDITIONAL FILE 1: SUPPLEMENTAL FIGURES

Integrated analysis of the aging brain transcriptome and proteome in tauopathy

Carl Grant Mangleburg , Timothy Wu, Hari K. Yalamanchili, Caiwei Guo, Yi-Chen Hsieh, Duc M. Duong, Eric B. Dammer, Philip L. De Jager, Nicholas T. Seyfried, Zhandong Liu, Joshua M. Shulman

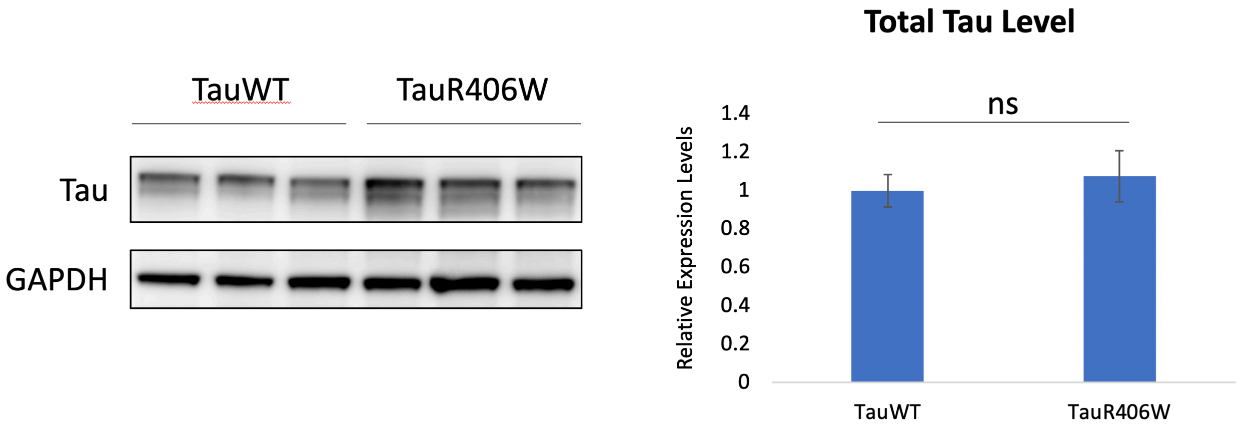

Figure S1. Human Tau protein is expressed at comparable levels in *elav>Tau*^{WT} and *elav>Tau*^{R406W} transgenic flies. Western blot quantification of Tau protein expression from 1-day-old animals (n=3 per genotype). Statistical analysis was performed using student's t-test.

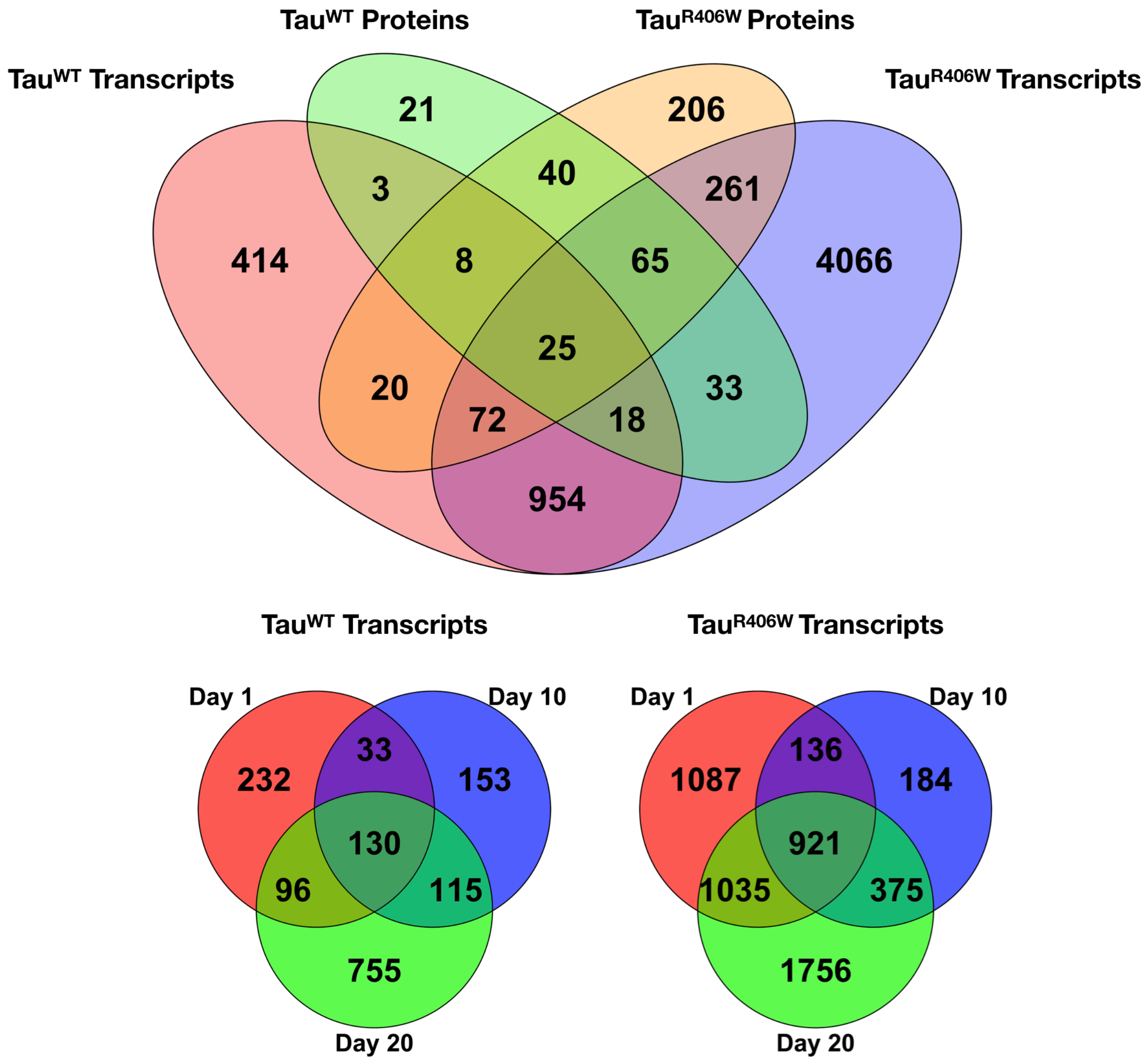

Figure S2. (Top**)** Venn diagram showing the number of differentially expressed genes shared between the transcriptome and proteomes of either *elav>Tau*^{WT} or *elav>Tau*^{R406W} transgenic flies, when compared with *elav* controls. Results of cross-sectional analyses stratified by age were combined (total of 1-, 10-, and 20-days). (Bottom) Venn diagrams showing overlaps of Tau^{WT}- (left) or Tau^{R406W}-induced differentially-expressed genes across the 3 ages evaluated (1-, 10-, and 20-days). See also Table 1 and Additional File 2: Table S1.

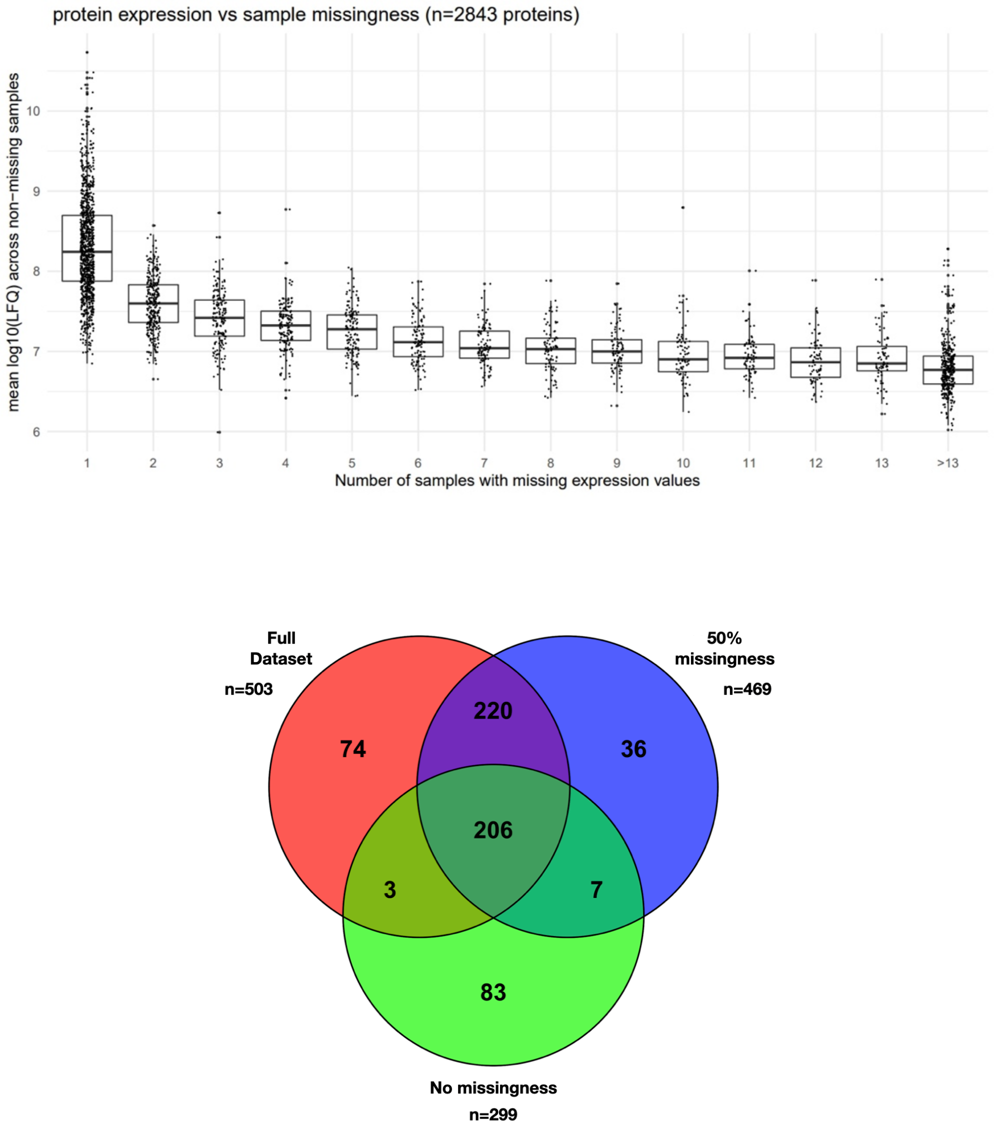

Figure S3. Potential impact of missingness on proteome analyses. (Top) Number of samples in which protein values are missing (out of total 27 samples) versus protein abundance. The plot highlights increased missingness for low abundance proteins. (Bottom) In order to evaluate the potential impact of missingness and imputation on our results, we repeated our analysis of age-adjusted differential protein expression in Tau^{R406W} vs control animals using either the *high-* (n=1,013 non-missing proteins) and *moderately-stringent* filtered datasets (n=2542 proteins with <50% missingness), and compared with the results from our fully-imputed dataset (n=2,723 proteins). Out of the 503 differentially expressed proteins identified in our complete, imputed dataset, 209 (42%) and 426 (85%) overlapped with the high and more moderately stringent comparison analyses, respectively. Overall, 33% (206 out of 629 total) differentially expressed proteins are shared across the 3 analyses. Detailed results of this sensitivity analysis are included in Additional File 1: Table S12.

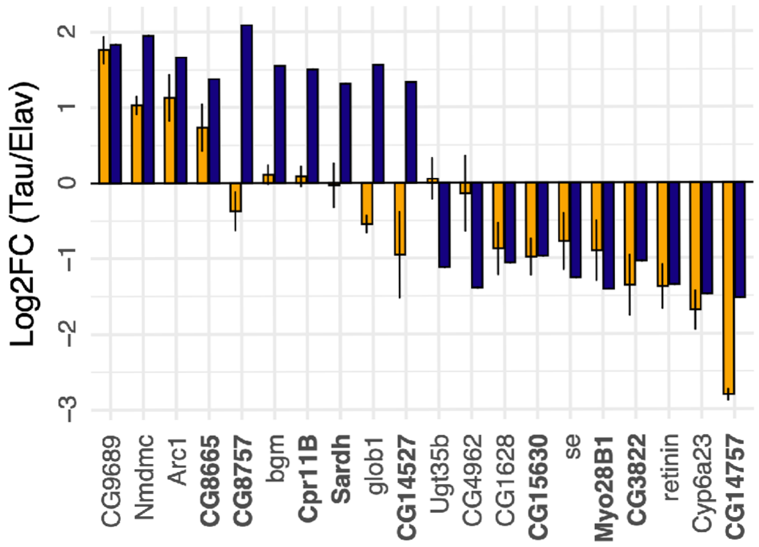

Gene	Forward Primer	Reverse Primer
CG8757	ATGGAGCGTTGGTGCAATAAA	GAGACTCAGACCACTGCGAAG
Nmdmc	GCACCCAACTAGCACACGA	CGACCATCTTGTTAGCCACATAC
CG9689	CACAGCCTGCAATGCACTC	GCTGGAAGAGCGATATGGTGG
Arc1	ATGGCCCAGCTTACACAGATG	GGAGAAGTTGCCTTTGCCTC
glob1	ACCAACAGATTCTGGAGCGG	TCCAAAGGAACATCGCGGAA
bgm	CATCGCCGGAATCTACACCAC	GCGTGAATCTTGTCCATTTGCT
Cpr11B	ATGCTGCGACCTCTGTTGAC	CCGTTGGCTAAGACGACCG
CG8665	AAGGCAGTCGAGAGGATATTTTG	CAAGACCTCAGGTAGTGCCAC
CG14527	AGGATGTACTCAACCAGGACAC	TGATGCGTTTATAGTTGCCACAG
Sardh	CTGTCACACACTGTATCACCTG	CGACGTGAATTAGCCAGCAG
CG14757	ATCTTCCACTGCCGGAGTATC	GCTTGCGCGATTGGTACAAAA
Cyp6a23	TGTGCCCCATCCAATATACGG	TAGTCCCGGAAGATCATCGCA
Myo28B1	GCCGCAAAATACTACCAGCG	ATTGCCTTCAGGACATCCCC
CG4962	ACTCCACCTCCACCTACCAT	CCCAGACCGTAGCTACCGTA
retinin	GAGCAACAGGTCTCCTCCG	GGTCAATACGGTTCGTAGTCCA
se	CACAGAGTCGCTACTGATTTGT	CGCTCGATTAGTAACTTGTCCTG
Ugt35b	CCGGGGCCATCACAGTATATT	GGAAACGCATTAACCGAGGTC
CG1628	TTTGAGCACATACCGCAAGGA	AATTTCTGGCATCCACCGTAG
CG3822	ATCAAGATCGGCGGTCTCTTC	TCAGTAAGCCACAAACCCTTTT
CG15630	GGCGAAATTATACGCGAGCAC	GGTGGCATTTTCCGTGAATGTA
Gapdh1	TAAATTCGACTCGACTCACGGT	CTCCACCACATACTCGGCTC
Rpl32	ATCGGTTACGGATCGAACAA	GACAATCTCCTTGCGCTTCT

Figure S4. Reverse transcription-quantitative PCR (RT-qPCR) validation of differentially expressed genes in 10-day-old Tau^{R406W} animals. mRNA was prepared from n=3 samples for each genotype (*Elav>Tau^{R406W}* or *Elav-GAL4* controls), each consisting of 100 fly head homogenates. Gene expression from RT-qPCR was normalized to *Rpl32* expression. PCR primers are indicated in the table. Fold-change (log_2-normalized) is shown based on RNA-seq (blue) or RT-qPCR (orange). Error bars denote +/- 1 standard error of the mean (SEM) across replicate samples. Overall, 15 out of 20 genes showed consistent differential expression. Genes in boldface showed significant differences in dCt (Delta Cycle threshold) between Tau^{R406W} vs. control samples (Welch's t-test, $p<0.05$).

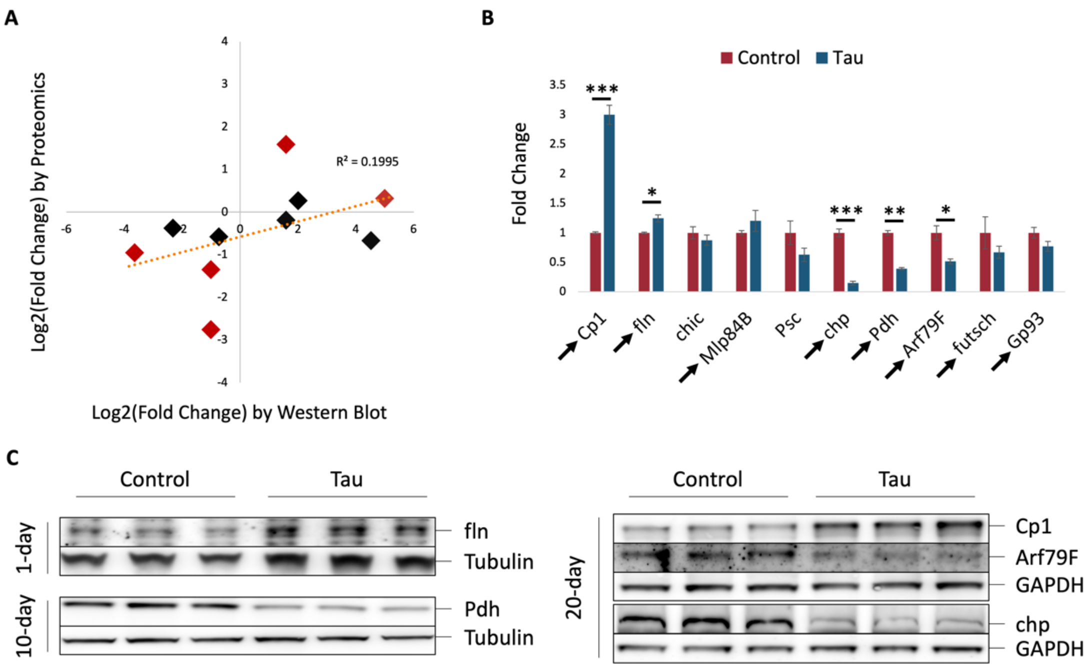

Figure S5. Western blot validation of differentially expressed genes from LC-MS/MS proteomics. (**A**) Correlation analysis of results from proteomics and western blot analysis using $\log_2$(fold change) values. Proteins with significant differential expression in Tau^{R406W} flies detected by western blotting are denoted in red. (**B**) Quantitation of western blots examining protein expression in control (*elav-GAL4*) vs. *elav>Tau*^{R406W} flies (n=3 samples per genotype). Proteins showing consistent direction of change based on LC-MS/MS and western blotting are indicated by arrows. Error bars denote mean± SEM. Statistical analyses based on two-tailed unpaired t tests. *, $p < 0.05$; **, $p < 0.01$; ***, $p < 0.001$. (**C**) Representative blots showing selected proteins with significant differential expression in Tau^{R406W} flies compared to age-matched control flies (fln: 1-day flies, Tubulin as the loading control; Pdh: 10-day flies, Tubulin as the loading control; Cp1, Arf79F, chp: 20-day flies, GAPDH as the loading control).

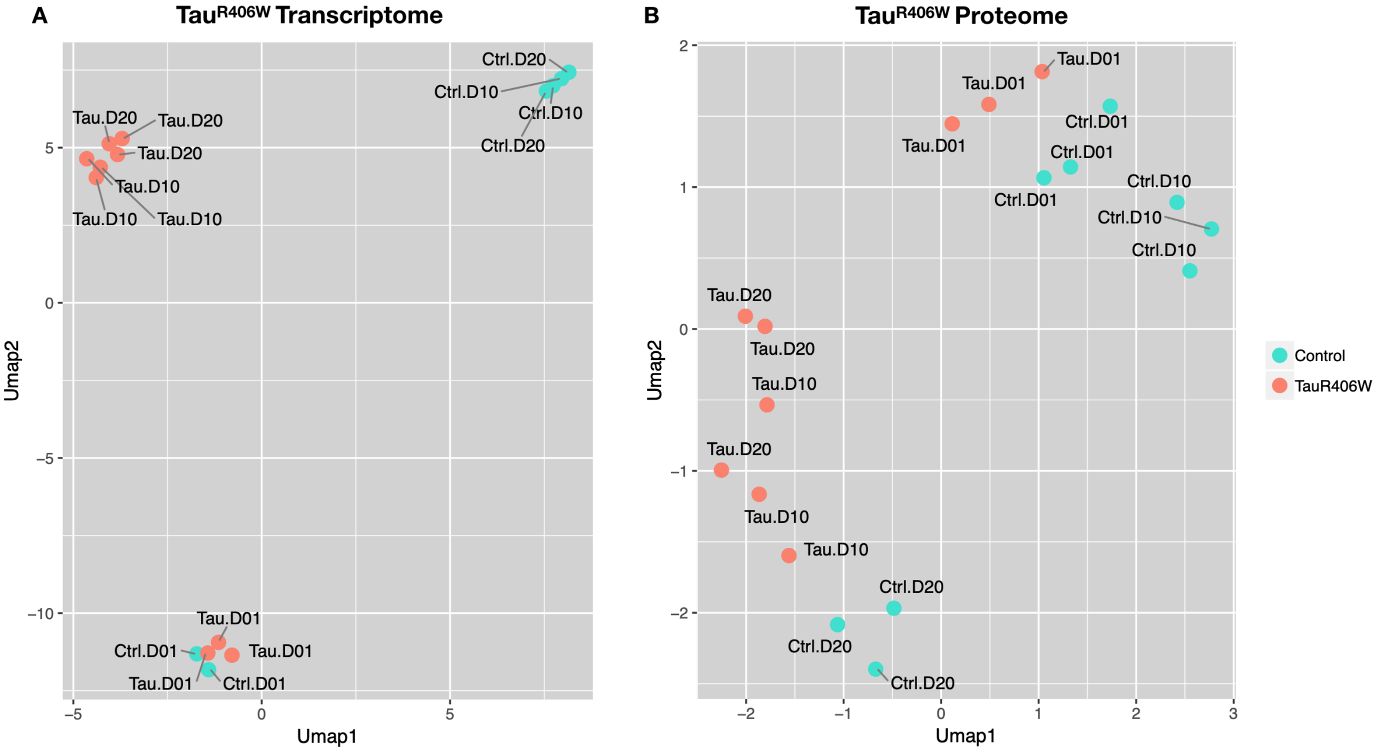

Figure S6. Age and Tau are major drivers of gene expression differences among samples. Uniform manifold approximation and projection (UMAP) of Tau^{R406W} transcriptome (A) and proteome (B) expression data. Raw counts were normalized for library size and log-transformed. *elav* control (Ctrl, blue) and *elav>Tau^{R406W}* (Tau, red) samples are shown, including from 1-, 10-, and 20-day old animals (D01, D10, D20, respectively). See also Fig. 1a, b.

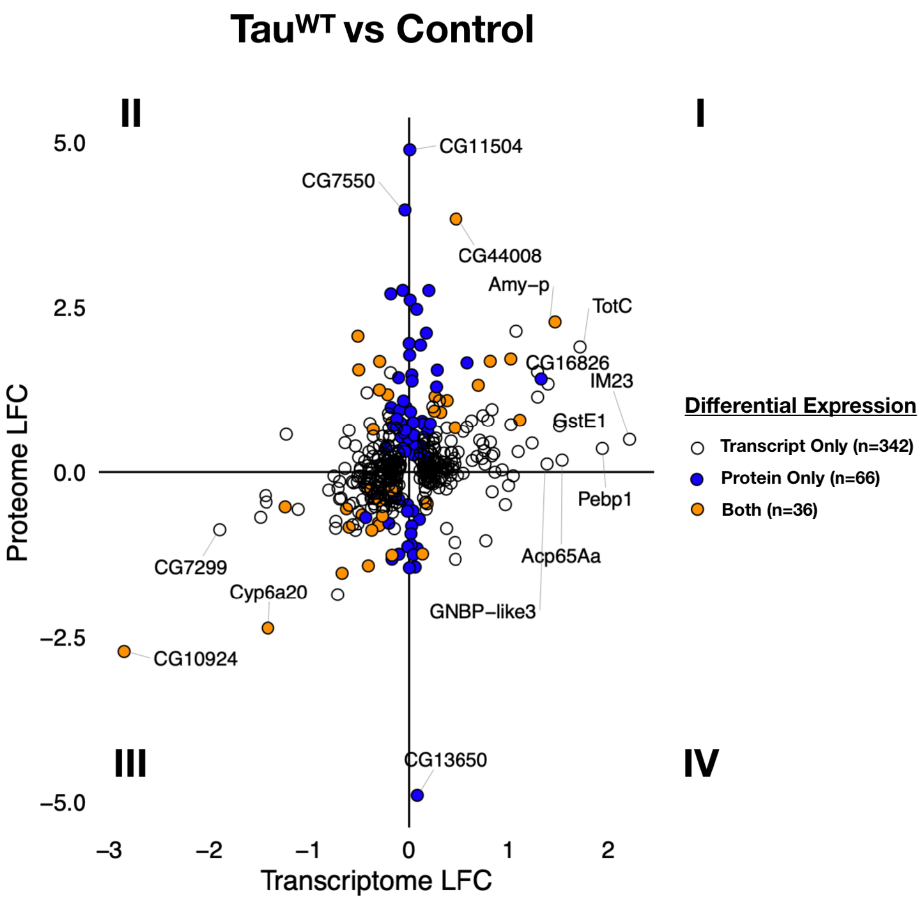

Differential Expression	Concordant (I + III)	Discordant (II + IV)
Transcripts	251 (66%)	127 (34%)
Proteins	63 (62%)	39 (38%)

Figure S7. Plot (top) showing Tau^{WT}-triggered $\log_2$ fold-change (LFC) in the transcriptome and proteome. The plot only includes those genes detected as both transcripts and proteins and also differentially expressed (n=446, FDR < 0.05), based on the joint regression model including longitudinal data and adjusting for age. Colors denote whether gene was differentially expressed in the transcriptome (unfilled), proteome (blue), or both (orange). Quadrants I and III include gene expression changes that are concordant (same direction) at the transcript and protein level; whereas quadrants II and IV depict discordant changes. A substantial proportion of differentially-expressed transcripts or proteins are discordant (table, bottom).

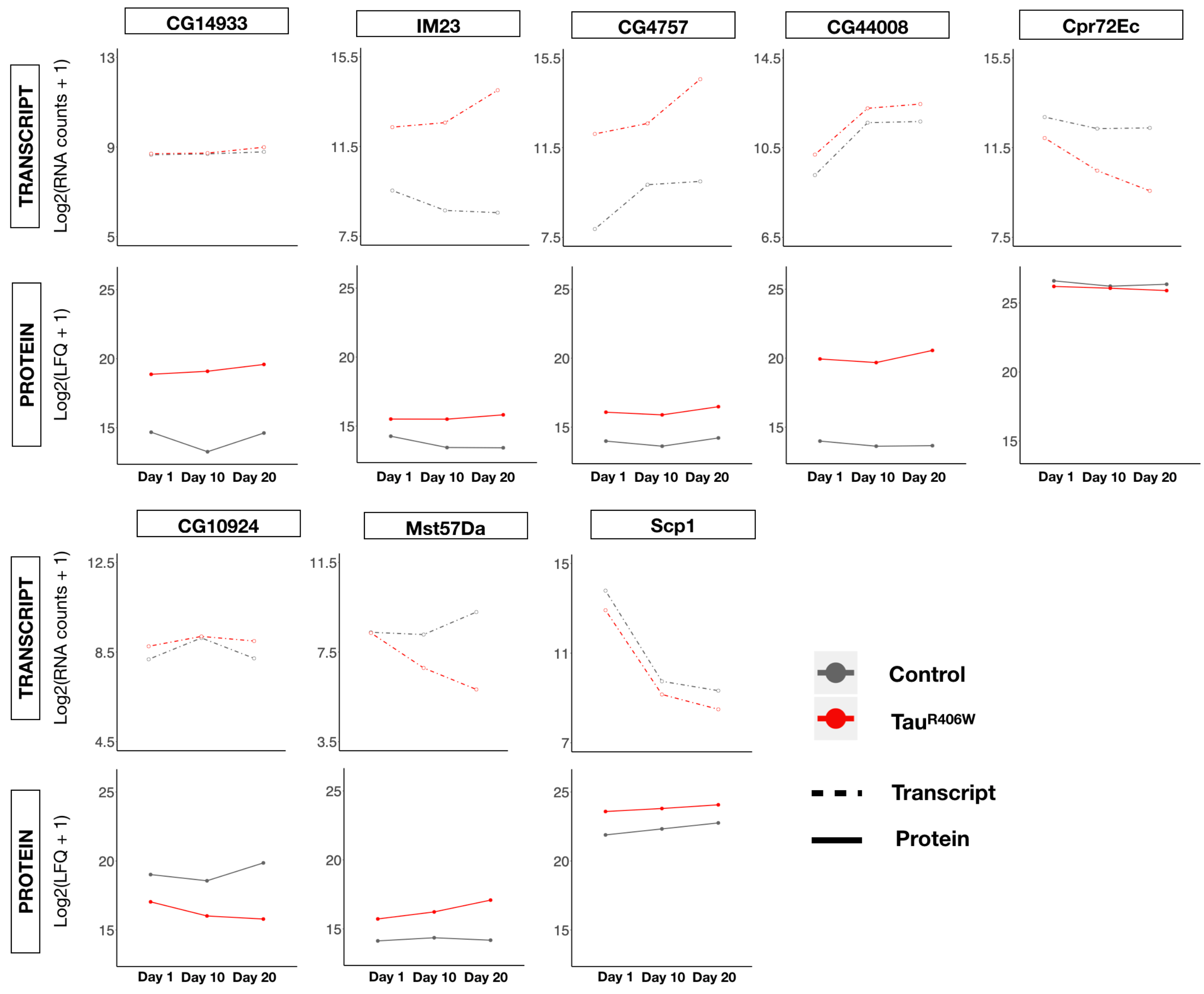

Figure S8. $\log_2$-transformed expression of selected genes in *elav>Tau*^{R406W} (Tau, red) and *elav* (Control, gray) is shown for transcriptome (depth normalized counts) and proteomes (normalized label-free quantification intensity (LFQ)). The selected genes are also noted in Fig. 1c. and are differentially expressed (FDR < 0.05) in both the transcriptome and proteome, except CG14933 and CG10924 which are protein-only, based on the joint regression model including all longitudinal data and adjusting for age.

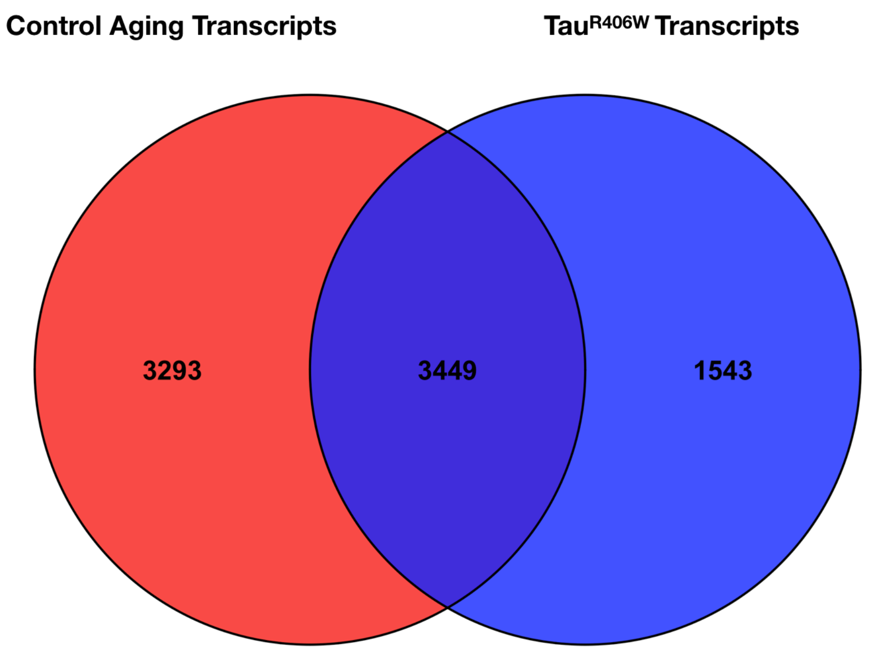

Figure S9. Venn diagram showing overlap between *elav>Tau^{R406W}*-induced (blue) differentially-expressed transcripts and aging-induced changes (red) detected from batch-matched *elav* controls. Tau^{R406W} -induced differential expression is based on the joint regression model including all longitudinal data and adjusting for age. The total number of aging-associated, differentially-expressed transcripts are based on the union of 3 comparisons (1- vs. 10-days, 10- vs. 20-days, and 1- vs. 20-days). See also Table 2 and Additional File 2: Tables S2, S3.

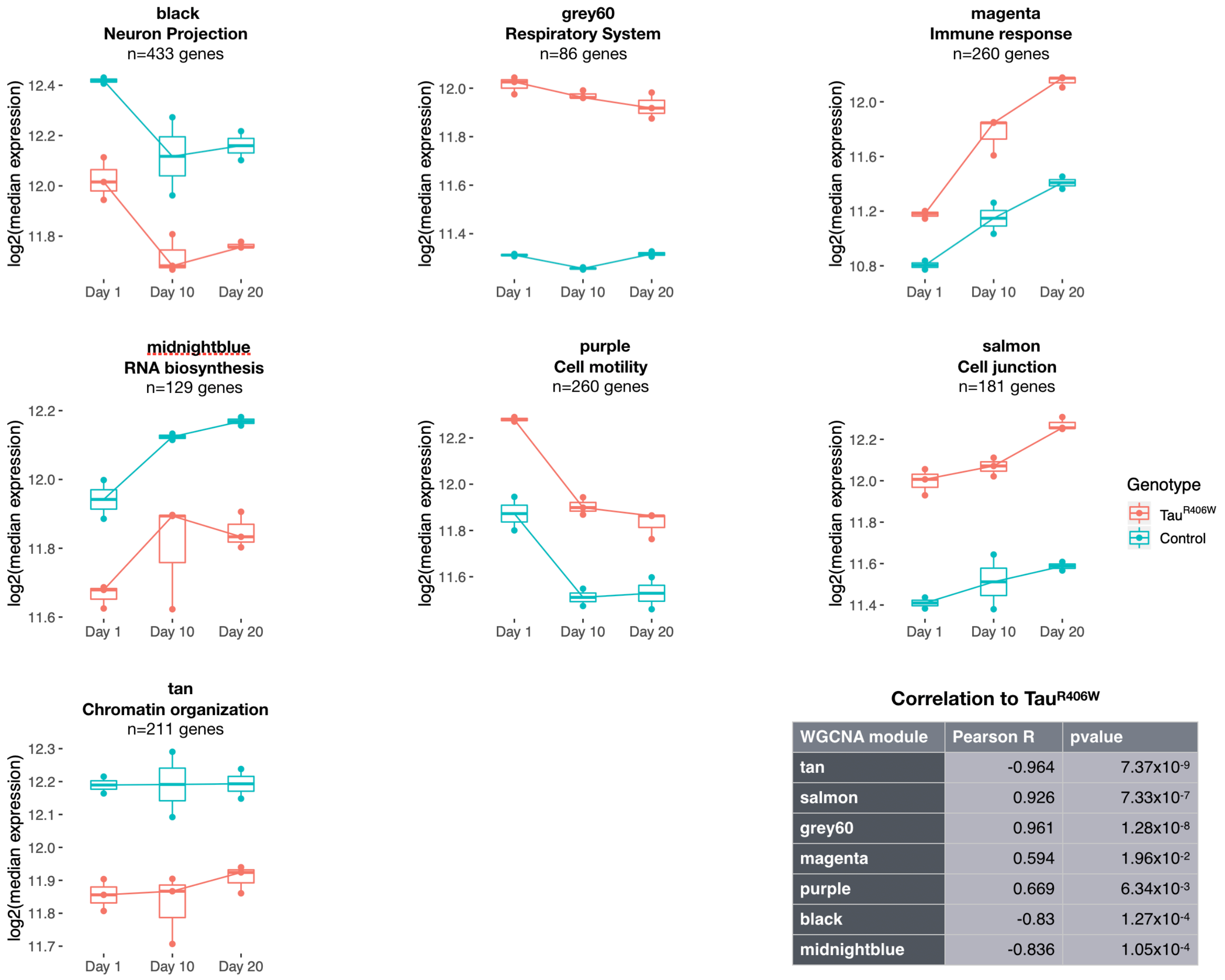

Correlation to Tau^{R406W}

WGCNA module	Pearson R	pvalue
tan	-0.964	7.37×10^{-9}
salmon	0.926	7.33×10^{-7}
grey60	0.961	1.28×10^{-8}
magenta	0.594	1.96×10^{-2}
purple	0.669	6.34×10^{-3}
black	-0.83	1.27×10^{-4}
midnightblue	-0.836	1.05×10^{-4}

Figure S10. Seven out of 15 WGCNA modules were significantly correlated with *elav>Tau*^{R406W} genotype as described in the methods. Boxplots show log2-transformed median expression of genes within each of these correlated modules, including *elav>Tau*^{R406W} (Tau, red) and *elav* (Control, blue). Clusters are annotated based on size and significantly enriched gene ontology terms. Pearson coefficients and p-values for module correlations to Tau^{R406W} genotype are also shown. See also Additional File 2: Tables S6, S7.

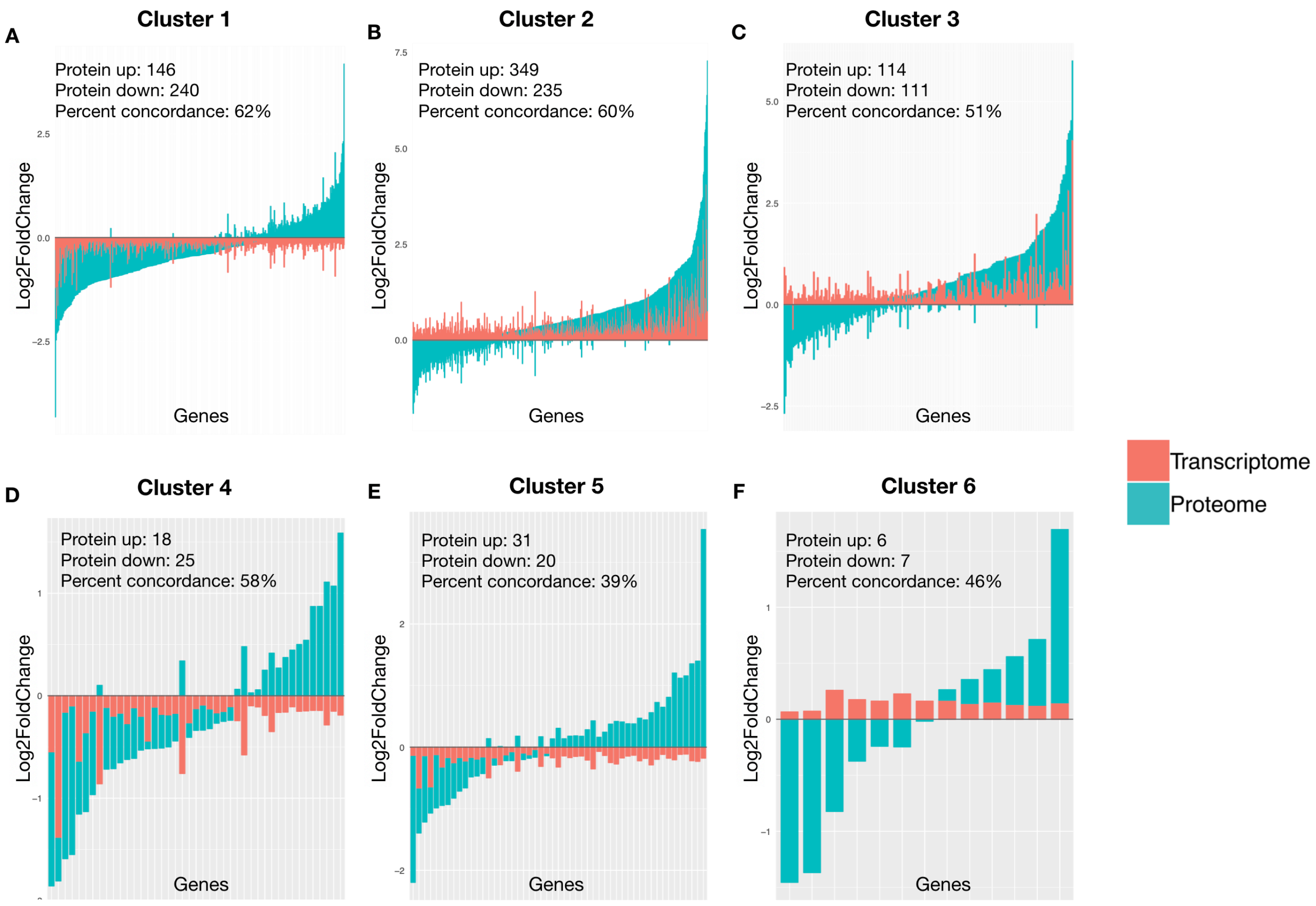

Figure S11. Integration of Tau-induced transcriptome and proteome changes in gene expression clusters. Unsupervised hierarchical clustering was performed on 4,992 differentially expressed transcripts (FDR < 0.05) in *elav>Tau*^{R406W} vs. *elav* controls, based on the joint regression model, including all longitudinal data and adjusting for age. Log2 fold-change of for gene members of the 6 resulting, mutually-exclusive clusters are shown. Stacked bar plots are restricted to those genes detected in both the transcriptome (red) and proteome (blue). The number of proteins that are either up- or down-regulated and the percent concordance between proteins and transcripts are noted. As a result of hierarchical clustering, transcripts within a given cluster have consistent direction of change (up or down). See also Fig. 3.

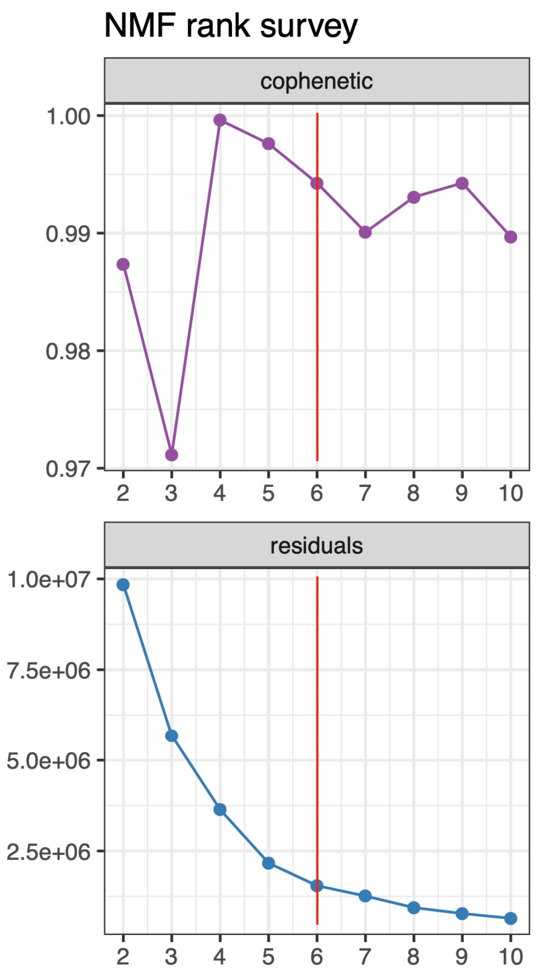

Figure S12. For determination of optimal dendrogram cuttoff (n=6, red line) for unsupervised hierarchical clustering, non-negative matrix factorization rank survey was performed, considering all differentially expressed transcript levels in *elav>Tau*^{R406W} vs. *elav* controls, based on the joint regression model, including all longitudinal data and adjusting for age. Either cophenetic correlation coefficients (top) or model residuals (bottom) are shown versus the cluster number (x-axis).

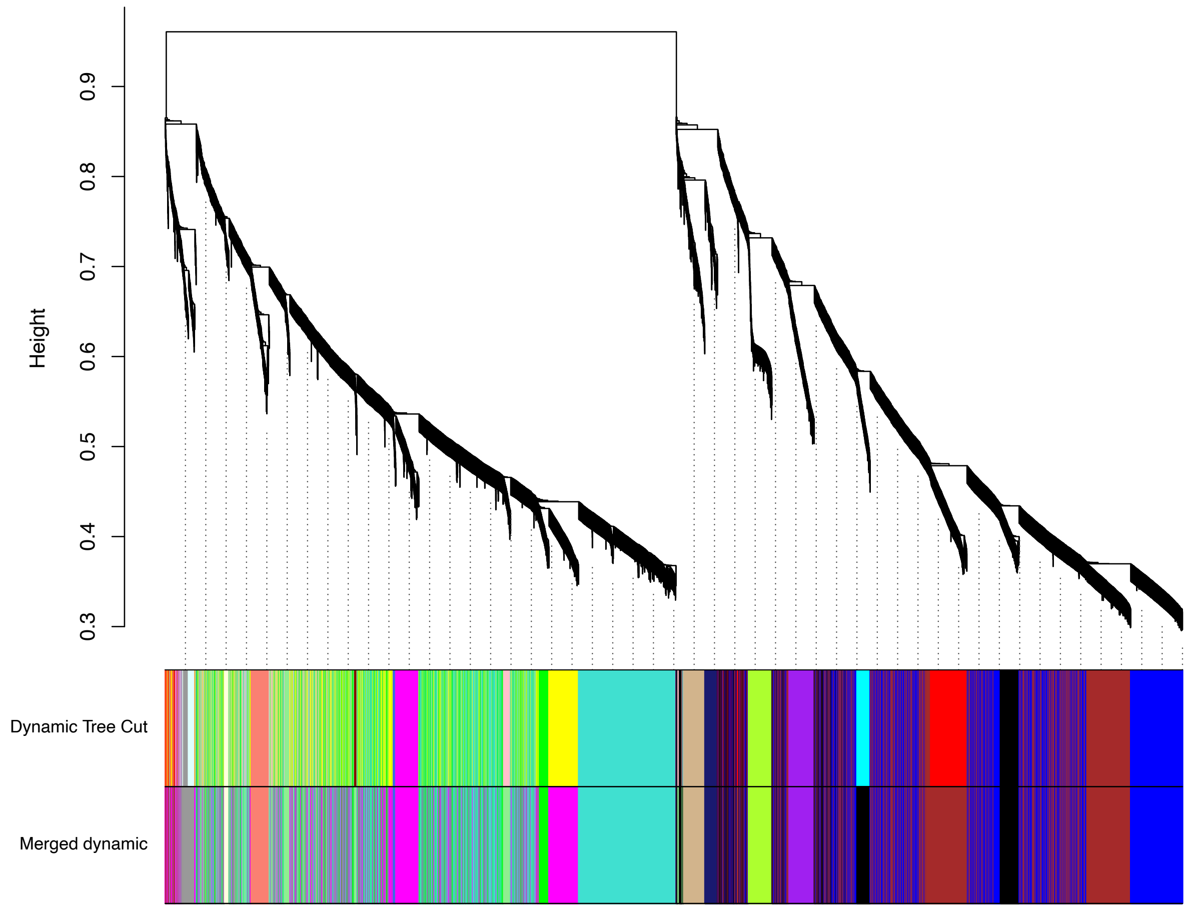

Figure S13. WGCNA cluster dendrogram showing tree heights/coexpression by normalized expression levels across all transcripts in *elav>Tau*^{R406W} and batch-matched *elav* controls. A tree height cut was made to generate the modules shown in the color strips. The dynamic tree cut color strip denotes the modules (labeled in colors) assigned from branches in the dendrogram. The merged dynamic color strip shows new module identities after combining closely related modules (determined by module eigengene with a 0.1 tree height cutoff).
